# Elastic Constants of Polymeric Fiber Composite Estimation Using Finite Element Method

**DOI:** 10.3390/polym16030354

**Published:** 2024-01-28

**Authors:** Calin Itu, Maria Luminita Scutaru, Sorin Vlase

**Affiliations:** 1 Department of Mechanical Engineering, Faculty of Mechanical Engineering, Transylvania University of Brasov, B-dul Eroilor 29, 500036 Brasov, Romania; calinitu@unitbv.ro; 2 Technical Sciences Academy of Romania, B-dul Dacia 26, 030167 Bucharest, Romania

**Keywords:** composite, fiber-reinforced, vibration, FEM analysis, material constants

## Abstract

Determining the properties of composite materials (knowing the properties of the component phases) is a primary objective in the design phase. Numerous methods have been developed to determine the elastic constants of a composite material. All these methods are laborious and require significant computing time. It is possible to make experimental measurements, but these too are expensive and time-consuming. In order to have a quick estimate of the value of the engineering constants of a new composite material (in our study a polymeric matrix reinforced with carbon fibers), this paper proposes a quick method for determining the homogenized material constants, using the finite element method (FEM). For this, the eigenfrequencies of a beam specimen manufactured by the studied composite material will be computed using FEM. The model will consider both phases of the composite, with the geometry and real size. The mechanical properties of the constituent’s material phases are known. With the help of this model, the torsional, longitudinal and transverse vibrations of the beam are studied. Based on the eigenvalues obtained by this calculation, it now is possible to quickly estimate the values of homogenized material constants required in the design. An example for a fiber-reinforced polymer composite material is provided in the paper.

## 1. Introduction

Currently, numerous methods are used to determine the elastic constants for a polymer composite material and there is rich literature in the field. Most of the methods are based on a theoretical model that involves knowledge of the stress and strain fields for arbitrary material loading. Other methods study particular cases of loading, but in the end, only upper and lower limits are obtained for the elastic constants of the material. This second class of method can lead to errors [1]. For example, in the cited paper, the case of an orthotropic composite and a transversely isotropic one is considered. Usually, such estimates, which consider particular cases of loading, are quite imprecise on certain ranges of component concentration [2,3,4,5]. Micromechanical models are mainly used to solve such problems. Some examples can be found in [6,7]. The obtained results agree very well with the research performed in [8]. For fiber-reinforced composites, a category of materials widely used in practice in the last decade, numerous studies have been carried out to determine these homogenized constants [9,10,11,12,13,14,15,16]. Different aspects related to solving the problem are presented in [17,18,19]. Other important methods are the experimental methods, but it is very clear that these are expensive and time-consuming.

In some studies, a representative volume element (RVE) is used to determine the homogenized elastic coefficients. The material is made up of a lot of such representative volume elements. At the microstructure level, composite materials have individual topologies and geometries, characterized by a wide variety. These properties will ultimately determine the behavior of the material as a whole. Based on the analysis of a single RVE or a group of RVEs, a method is proposed that allows for the consideration of the material as isotropic or transversally isotropic or orthotropic material, depending on the concrete situation (defined by the individual geometry and topology). Different microstructural patterns must obviously be considered for each type of composite material; for example, a composite material with short fibers is analyzed in [20]. The FEM, successfully used to calculate the mechanical properties of composite materials using static models, can also be used in another approach to the problem, namely to determine these properties using dynamic models by analyzing the vibrations of the studied systems. The advantage of the method is that it allows for the analysis of a very wide class of materials, with very different topologies and geometries. Thus, materials with very different properties can be analyzed with this method. Experimental verifications proved the validity of using the FEM [20]. The method proves to be an extremely useful tool for studying the mechanical properties of composites [21].

It is possible to use the FEM to study the influence of temperature or other factors, such as moisture absorption, on the mechanical behavior of composite materials. For unidirectional graphite fiber-reinforced composites, an analysis is presented in [22]. A similar model is used in [23,24] to calculate the elastic constants, and their dependence on the constituent phases of the composite is analyzed. In these papers, the authors deal with the determination of the elastic constants for a composite with transverse isotropic behavior. Young’s modulus is generally frequency-dependent. In [25], using system identification techniques, the method of obtaining these values experimentally, with maximum precision, is presented. Some examples are presented for brass, copper, plexiglass and PVC.

Hose pipes are usually made of rubber with metal braids. They have many applications, and knowledge of the mechanical properties is an important desideratum for the designer. Because the manufacturing method is complex, it is difficult to estimate, by calculation, these properties. An additional check was performed by performing a calculation with finite elements. The determination of Young’s modulus and damping ratios for fiber-metal laminated cantilever beams is presented in [26]. To verify that the results are correct, a theoretical model was also studied, obtaining a good agreement with the experimental results. To determine the properties of some materials when insufficient data are available, such as the determination of the mechanical properties of some wood species, a finite element analysis is proposed in [27]. Wood is an orthotropic material, so it is necessary to know the Young’s modulus in two directions, as well as the shear modulus and Poisson’s ratios. Finite element analysis with values of these quantities were attempted until the experimentally obtained results matched the natural frequencies of the structure calculated by using the FEM. Once these elastic constants are determined, more complex calculations can now be made for structures made with these materials. Evaluating the properties of complex materials, such as wood, is difficult to achieve using classic techniques. That is why the determination of the natural frequencies and the natural vibration modes can become valuable tools for the analysis of such unconventional materials [28]. Transverse and torsional vibration measurements to determine the interlaminar shear modulus for cardboard are presented in [29]. Timoshenko’s vibration theory was used together with the FEM to verify the results.

In general, to determine the mechanical properties of new, non-conventional materials, for which there is not enough data, experimental vibration measurements are used, from which the natural frequencies are obtained, which help us determine different engineering constants [30,31,32,33,34,35].

In particular, due to their use in a wide range of applications, cylindrical fiber-reinforced polymer composites have been intensively used. As a result, they have been studied intensively. In what follows, we present a series of studies with more interesting applied results, from the rich and specialized literature. Among the many materials that have been reinforced with fibers is concrete, which has an extraordinary use in practice. The base material is reinforced with cylindrical iron fibers. And in this case, the FEM proved to be an extremely useful method to predict the properties of the manufactured material.

Thus, for a practical application, the stress and strain fields were obtained, which were then used in the classical theory of homogenization to calculate the two elastic constants necessary to define the behavior of the homogenized material. The values of the engineering constants were obtained in good agreement with the values that were determined using other calculation methods. Applications of natural fiber-reinforced composite materials have become extremely popular in modern industry, especially because such materials can be recycled more easily. Natural fibers have a viscoelastic behavior, and, as a result, studying them is more complicated, involving methods that take into account the time parameter, which leads to numerous calculations [36]. But natural fibers have superior specific properties, exceptional ecological characteristics and a low price. The mentioned paper uses a classic homogenization procedure and proposes an analytical model for the study of such materials. The obtained results are verified by experimental measurements, which prove to be in excellent agreement between them (applying the proposed model) and the experimental results.

A method developed for the study of polymer composites, with viscoelastic behavior, reinforced with glass or carbon fibers, is developed in [37]. The behavior of this material is determined by the properties of the matrix and is viscoelastic. So, the time factor also appears in the development of the method because in this case, the flow of the studied material at higher temperatures must also be taken into account. The results obtained in the paper based on the proposed model are then verified experimentally. Other studies in the field, close to the object of the current paper, are presented in [38,39].

The novelty of the work consists in the use of the FEM instead of experimental measurements to determine the natural frequencies of a straight beam, fixed at both ends. The beam is made of a polymeric composite reinforced with carbon fibers. Based on the classical theory of the beam, knowing the eigenfrequencies thus determined, the values of some of the engineering constants of the homogenized material are determined.

In the paper, using classical models for the analysis of the vibrations of a classical beam, made of a composite material, the FEM was used to determine some of the elastic constants of the material. The method has an advantage over experimental methods, being cheaper and easier to apply. Also, the time required for modeling and obtaining the results is reduced. The method is applied to a composite reinforced with unidirectional cylindrical carbon fibers. This method is useful because it can be applied to a large class of composite materials to obtain the elastic characteristics of the composite material. A cylindrical fiber-reinforced composite is analyzed in the study, but the method can be easily extended to other types of composites with a more complicated topology and geometry. At the same time, materials composed of more than two phases can be considered.

## 2. Materials and Methods

**Figure 1:**
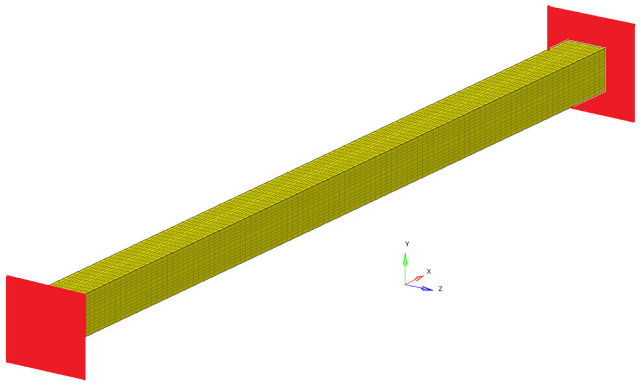
The clamped beam.

In the following, the main results from the classical theory of straight beam, clamped at both ends, regarding torsional, longitudinal and transverse vibrations are briefly presented. The relationships obtained allowed for the obtainment of some of the elastic constants of the studied materials. Using the FEM, it is possible to obtain the eigenfrequency for the beam and by inspecting the representation of the eigenmodes of vibration, provided by the software used, it is possible to identify the modes due to the torsional vibration, axial vibration, and transverse vibration. In each case, using the relations known from the classic beam analysis, some of the elastic constants of the homogenized material can be determined. Figure 1 shows the beam discretized into finite elements and clamped at the ends. Using this model, performed with Altair 2020, it is possible to obtain the eigenfrequencies of the beam made by a homogenized material. For the case of a polymeric material, the calculations are made and the results are presented in the Section 3.

(a) Torsional vibration. The free torsional vibrations of a beam are given by the differential equation, written at the distance *x* from the left end of the beam and in the moment [40,41,42,43,44,45,46,47,48]:

(1) $$GI_{p}\frac{\partial^{2}\phi }{\partial x^{2}}=J\frac{\partial^{2}\phi }{\partial t^{2}}\;,$$

where G is shear modulus, (interesting in our study), $I_{p}$ is the inertia moment of the area, *J* is the unitary mass moment of inertia, and ϕ the rotation angle of the current area. For a homogeneous, continuous beam, we have $J=\rho I_{p}$ and Equation (1) becomes:

(2) $$\frac{\partial^{2}\phi }{\partial x^{2}}=\frac{\rho }{G}\frac{\partial^{2}\phi }{\partial t^{2}}\;$$

Applying the classic theory concerning the torsional vibration of a beam, it obtains the eigenfrequencies of the homogenized beam:

(3) $$p_{n}=2\pi \nu_{n}=\frac{n\pi }{l}\sqrt{\frac{G}{\rho }}\;;\;n=1,2,3,\ldots \ldots$$

So, if an eigenvalue is known, the shear modulus can be determined for the material of the beam:

(4) $$G=\frac{p_{n}^{2}\;l^{2}\rho }{n^{2}\pi^{2}}=\frac{4\nu_{n}^{2}\;l^{2}\rho }{n^{2}}\;;\;n=1,2,3,\ldots \ldots$$

From Equation (4), the result is that between different eigenfrequencies there are the relations:

(5) $$\nu_{1}\;=\frac{\nu_{2}\;}{2}=\frac{\nu_{3}\;}{3}=\ldots \ldots =\frac{\nu_{n}\;}{n}\;;\;n=1,2,3,\ldots \ldots$$

which can be used to verify how good the obtained results are. So, with the FEM, more values are obtained for the eigenfrequencies, which give us more values for *G*. In this case, an average can be made for all these values to obtain a precise value for *G*.

(b) Longitudinal vibration. The differential equations that describe the longitudinal vibration are:

(6) $$\frac{\partial^{2}u}{\partial x^{2}}=\frac{\rho }{E}\frac{\partial^{2}u}{\partial t^{2}}\;.$$

Following the same operations as in case a), the eigenfrequencies are given by the relation:

(7) $$p_{n}=2\pi \nu_{n}=\frac{n\pi }{l}\sqrt{\frac{E}{\rho }}\;;\;n=1,2,3,\ldots \ldots$$

So, if an eigenvalue is known, the axial Young’s modulus can be determined for the material of the beam:

(8) $$E=\frac{p_{n}^{2}\;l^{2}\rho }{n^{2}\pi^{2}}=\frac{4\nu_{n}^{2}\;l^{2}\rho }{n^{2}}\;;\;n=1,2,3,\ldots \ldots$$

Relation (5) also remains valid for the longitudinal vibration.

(c) Transverse vibration. If we consider the transverse vibrations of the beam, the differential equation that describes these vibrations is:

(9) $$\frac{\partial^{4}v}{\partial x^{4}}+\frac{\rho A}{E_{z}I_{z}}\frac{\partial^{2}v}{\partial t^{2}}=0.$$

With the notation:

(10) $$p^{2}\frac{\rho A}{EI_{z}}=\alpha^{4}.$$

The eigenfrequencies are given using the relation:

(11) $$p_{n}=2\pi \nu_{n}=\alpha_{n}^{2}\sqrt{\frac{E_{z}I_{z}}{\rho A}}\;;\;n=1,2,3,\ldots \ldots$$

where $\alpha_{n}$ are (when both ends are clamped) the solutions of the transcendental equation:

(12) coshαl cosαl=1.

The first ten solutions β=αl of the transcendent equation coshβ cosβ=1 are:

4.7300; 7.8532; 10.9956; 14.1371; 17.2787; 20.4203; 23.5619; 26.7035; 29.8451; 32.9867 and will be used in the further considerations. The constant $\alpha_{n}$ will be:

(13) $$\alpha_{n}=\frac{\beta }{l}.$$

So, if an eigenvalue is known, the transversal Young’s modulus can be determined with the relation:

(14) $$E_{z}=\frac{p_{n}^{2}\;\rho A}{\alpha_{n}^{4}I_{z}}=\frac{4\pi^{2}\nu_{n}^{2}\;l^{4}\rho A}{\beta_{n}^{4}I_{z}}\;;\;n=1,2,3,\ldots \ldots$$

Using the presented results, three of the five elastic constants of an epoxy matrix reinforced with cylindrical fibers can be determined if the eigenfrequencies of the beam are calculated with the FEM.

## 3. Results

In the paper, a finite element analysis was performed of a straight beam, made of a composite made of a polymeric matrix reinforced with carbon fibers. The beam’s eigenfrequencies were determined. By studying the eigenmodes of movement, the eigenmodes due to transverse, longitudinal and torsional vibrations were identified. Based on the previously presented classic models that link the elastic constants to their eigenfrequencies, some of the elastic constants of the homogenized material were determined. The proposed method facilitates the calculation of these engineering constants, for which there are very laborious and time-consuming analytical determination methods [49].

Within the studied example, the eigenfrequencies and the eigenmodes were determined. Among these eigenmodes of movement, it was found that two describe the longitudinal vibrations of the beam, eight describe the torsional vibrations and ten describe the transverse vibrations. Three types of vibrations were analyzed. Based on the numerical results obtained with the FEM and taking into account the exact formulas known for the vibrations of the straight beam, the longitudinal Young’s modulus and the transverse and shear moduli were determined [50,51,52,53]. The beam is discretized using three-dimensional hexahedral finite elements with eight nodes. Each node involved 3 degrees of freedom (DOFs), which are displacements in the X, Y and Z directions (considered in the global coordinate system). Thus, the behavior of an element is defined by 24 DOFs. The details of the formulation can be found from reference [51].

**Figure 2:**
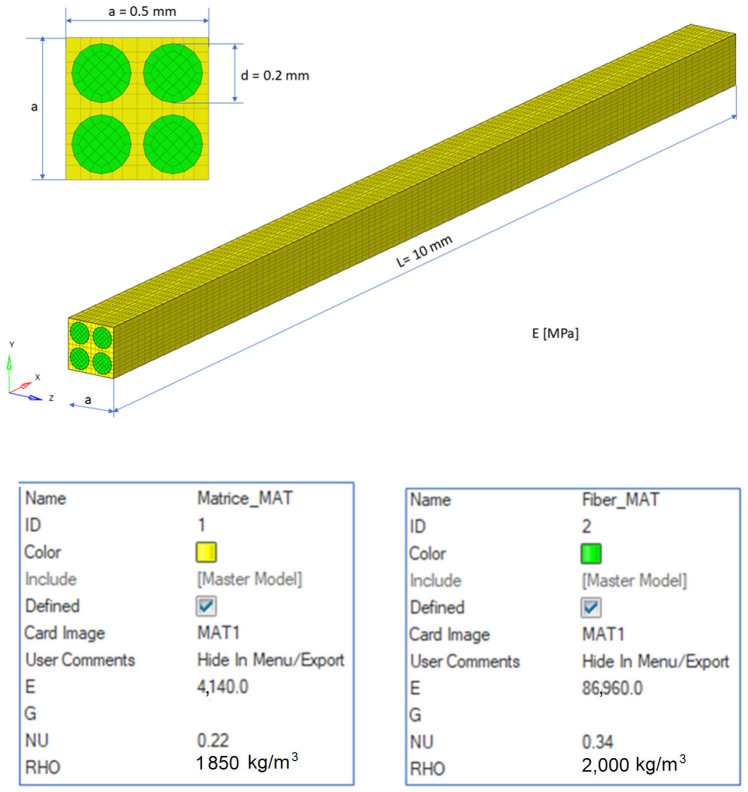
The composite material.

If the obtained results are analyzed and the eigenmodes are represented, the modes for torsional, longitudinal and transverse vibrations can be easily identified. We analyzed them in the following way to determine the elastic constants: In Figure 2, the FEM considered is presented. It is considered a simplified model with four carbon fibers, cylindrical and parallel incorporated in a polymeric epoxy matrix. The dimensions of the specimen are presented in Figure 2. The Young’s modulus for the carbon fiber is 86.960 GPa and for the matrix is 4.140 GPa. The density of the materials is 1850 kg/m^3^ for the matrix and 2000 kg/m^3^ for the carbon fiber. The Poisson ratio is 0.22 and 0.34for the matrix and carbon. These data were used for the model presented in Figure 2, where the torsional, longitudinal and transverse vibrations of this basic beam are analyzed. If we analyze the eigenmodes of vibration, these three modes of vibration can be identified. The symmetry of the bar allows an easy analysis of such a bar. Obviously, in more complicated cases, this is no longer possible. But precisely this simplicity was used in the work in order to quickly obtain estimates of some mechanical constants.

It is easy to observe that the eigenmodes 3, 5, 8, 11, 13, 15, 18 and 20 describe the torsional vibrations of the beam. Table 1 shows the torsional eigenfrequencies $\nu_{n}$ ($p_{n}=2\pi \nu_{n}$) and the corresponding eigenmodes. Based on Equation (4) previously introduced, the shear modulus can be calculated. It results:

(15) $$G=\frac{4\nu_{n}^{2}\;l^{2}\rho }{n^{2}\pi^{2}}\;;\;n=1,2,3,\ldots \ldots$$

So, knowing the eigenfrequency, the number of the eigenmode with this frequency and the length and the density of the homogenized material, it is possible to obtain the shear modulus. If the density of the fiber is $\rho_{f}$, the density of the matrix is $\rho_{m}$, the ratio of the fiber is $\nu_{f}$, the ratio of the matrix is $\nu_{m}=1-\nu_{f}$ and the density of the homogenized material is:

(16) $$\rho =\nu_{f}\rho_{f}+\nu_{m}\rho_{m}.$$

Modes 9 and 13 can be identified between the eigenmodes as being the eigenmodes that describe the longitudinal vibrations of the beam. Table 2 shows these eigenmodes and the longitudinal eigenfrequencies of vibration. Considering the classic theory of beams and based on Equation (8) offered by this theory, the longitudinal Young’s modulus can be determined. So, if the eigenfrequency $\nu_{n}$, the length *l* of the specimen, the density of the homogenized material and the ρ si number of the eigenmode *n* are known, the longitudinal Young’s modulus is obtained:

(17) $$E_{L}=\frac{4\nu_{n}^{2}\;l^{2}\rho }{n^{2}\pi^{2}}\;;\;n=1,2,3,\ldots \ldots$$

The modes 1, 2, 4, 6, 7, 10, 12, 14, 17 and 19 considered in our analysis are the eigenmodes that define the transverse vibrations of the beam. Table 3 shows the torsional eigenpulses and the corresponding eigenmodes. Using the solutions of Equation (14) previously obtained, it is possible to obtain the transverse Young’s modulus *E_z_*:

(18) $$E_{z}=\frac{4\pi^{2}\nu_{n}^{2}\;\rho A}{\alpha_{n}^{4}I_{z}}=\frac{4\pi^{2}\nu_{n}^{2}\;l^{4}\rho A}{\beta^{4}I_{z}}\;;\;n=1,2,3,\ldots \ldots$$

In Equation (18), *A* represents the area of the studied specimen and *I_z_* represents the moment of inertia of this area and β is the solution of Equation (12). Table 3 shows some of the transverse eigenfrequencies and the corresponding eigenmodes. Using the solutions of Equation (14) previously obtained, it is possible to obtain the transverse Young’s modulus *E_z_*. We considered the first three eigenfrequencies for the study. The value obtained after the calculus is 35.2963 GPa.

## 4. Discussion

The engineering constants of a polymer composite material, reinforced with cylindrical fibers, can be determined by analytical methods, which are generally very laborious and which assume, for the more precise methods, the determination of the field of stresses and deformations in the composite material. Experimental methods can also be used, which can give very good results but obviously involve high costs and lots of time. In the present work, using the classical beam theories to determine the natural frequencies and then the FEM and then comparing them with the simple formulas used in the classical theory, some of the material constants can be determined. The procedure has the advantage of simplicity and the possibility to obtain fast and accurate estimates in the design process. Considering a large number of eigenfrequencies can improve the estimation accuracy [40,41,42].

The method presented in the paper is correct if we assume a straight beam. Errors that may occur may be due to the FEM in particular, but at this point, the method is sufficiently accurate to meet engineering needs.

The problem of determining material constants for different composites and especially for polymer composites reinforced with cylindrical fibers distributed in parallel has been a subject intensively studied over time by researchers, due to the multiple applications of composites. Several analytical models have been proposed to solve the problem. The theory of homogenization involves the determination of the stress and strain fields. Also, the micromechanical model ultimately leads to the same problem of determining stress and strain. Methods have also been proposed that consider particular loading situations. In this case, however, upper or lower bounds were obtained for the elastic constants, which are sometimes far from the real values [7,8,9,10,11,12,13]. In the paper, the vibration behavior of the resulting material was studied, with the values obtained after the calculation being able to be related to the values of some of the important constants of the material such as axial tensile modulus, transverse tensile modulus, axial shear modulus, transverse shear modulus, axial Poisson ‘s ratio, transverse Poisson’s ratio or bulk modulus (depending on the resulting material, homogeneous and isotropic, transversely isotropic and orthotropic) [42].

Obviously, more complex situations involving thermal effects, humidity, etc., can be studied, which can no longer be neglected. In this case, the classical beam models that also take these effects into account must be considered. Obviously, in these cases, additional parameters may appear that must be taken into account in the calculations. For each of these special cases, the appropriate model must be considered and the appropriate calculations conducted.

## 5. Conclusions

The calculation method presented gives us a rapid estimate of the value of the engineering constants using the FEM to determine the eigenfrequencies of a simple beam specimen, clamped at both ends, made by a polymeric material reinforced with cylindrical carbon fibers. The material constants of the constituent phases of the studied composite are known. Using classical beam theory, some of the homogenized material constants are computed. This method is very simple and easy to apply. At the same time, the time required to use the procedure is short. These estimates are affected by the approximations that are made in classical models of the beam and by the errors that occur when using the FEM. The procedure offers a relatively simple and fast method of estimating the elastic constants of the composite material as a whole and can represent a very good solution in the design phase of a composite. For the polymer composites reinforced with carbon fibers, studied in the paper, the results obtained are similar to results previously obtained by other methods and verified experimentally, presented in the Introduction [40,41,42].

A more complex situation can be considered when it is necessary to take into account humidity, temperature or other factors. The proposed method is also effective in these cases. The use of the FEM is the same, and the difference occurs when considering the vibration of beams taking into account other influences, which depend on a parameter. For all new models, the FEM must be adapted so that it is possible to determine the elastic constant under these circumstances. The study of these new models may be the objectives of future studies.

## Figures and Tables

**Table 1:** Eigenfrequencies of the torsional vibration.

Mode No.	$\mathrm{Eigenfrequency}\;\nu_{n}$ [Hz]	Representation	$\mathrm{Transverse}\;\mathrm{Shear}\;\mathrm{Modulus}\;G=\frac{4\nu_{n}^{2}\;l^{2}\rho }{n^{2}\pi^{2}}$ [GPa]
3	82,288.62	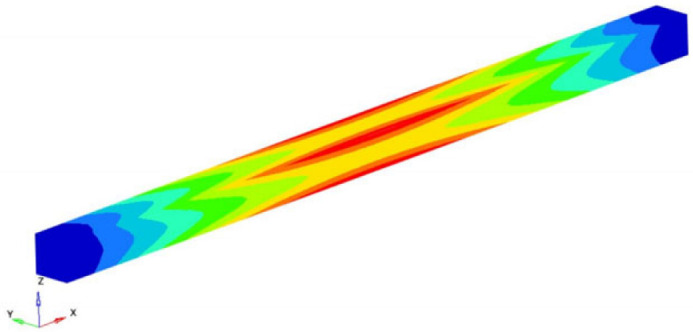	5.2129
5	164,757.2	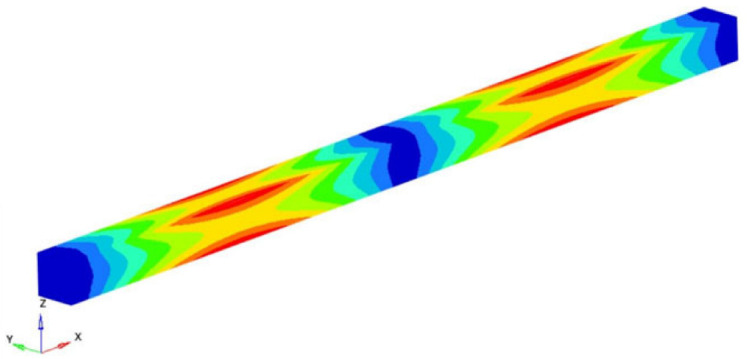	5.2243
8	247,584.1	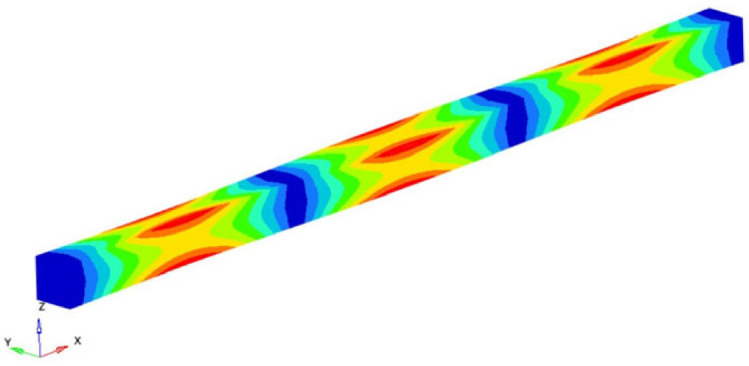	5.2432
Average shear modulus (GPa)			5.226834

**Table 2:** The eigenfrequencies of the longitudinal vibration.

Mode No.	Eigenfrequency [Hz]	Representation	$\mathrm{Longitudinal}\;\mathrm{Young}’s\;\mathrm{Modulus}\;E_{L}=\frac{4\nu_{n}^{2}\;l^{2}\rho }{n^{2}\pi^{2}}$; *E_L_* [GPa]
9	250,458.3	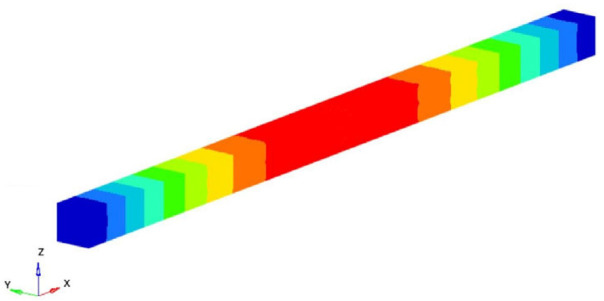	48.2915
16	500,238.9	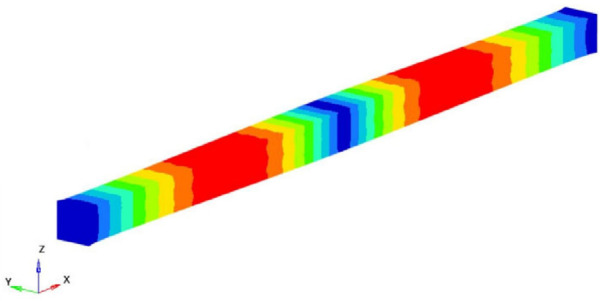	48.1609
Average longitudinal Young’s modulus E (GPa)			48.2262

**Table 3:** The eigenfrequencies of the transverse vibration.

Mode No.	Eigenfrequency [Hz]	Representation	$x=\alpha_{n}l$	$E_{z}=\frac{4\pi^{2}\nu_{n}^{2}\;l^{4}\rho A}{\beta^{4}I_{z}}$ [GPa]
1	22,159.00	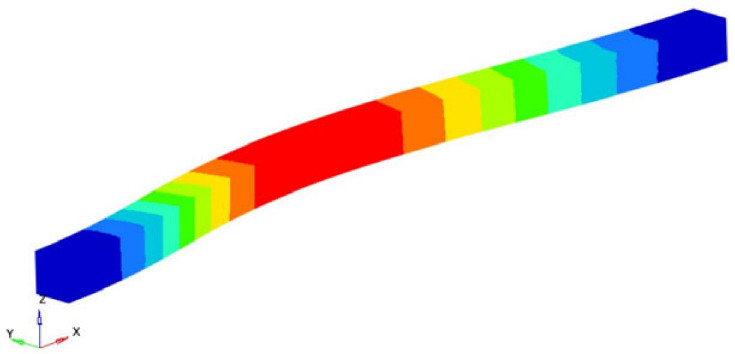	4.730040744862704	35.7752
2	55,787.45	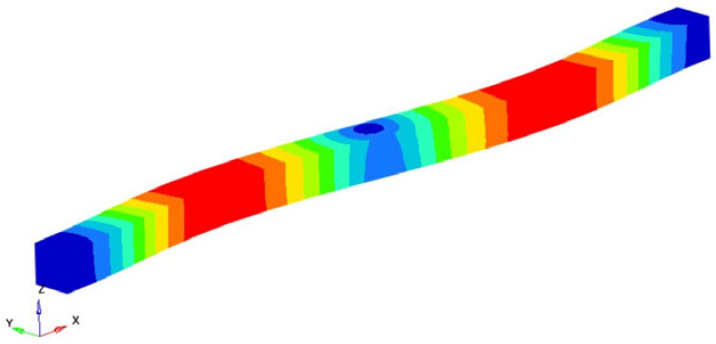	7.8532046240958376	34.5044
4	116,467.3	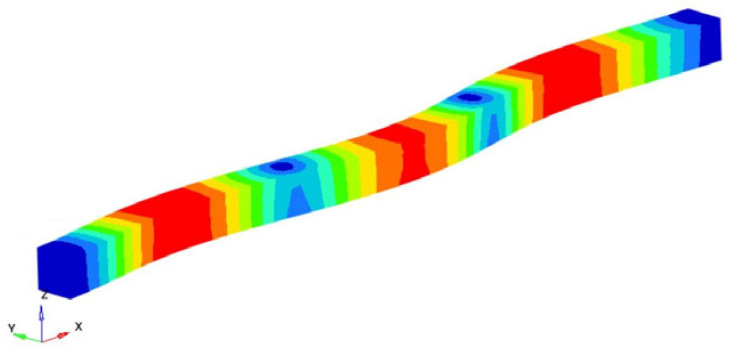	10.995607838001671	35.6093
Average transversal modulus (GPa)				35.2963

## Data Availability

Data are contained within the article.
